# Functional traits' annual variation exceeds nitrogen‐driven variation in grassland plant species

**DOI:** 10.1002/ecy.3886

**Published:** 2022-12-07

**Authors:** George R. Wheeler, Chad E. Brassil, Johannes M. H. Knops

**Affiliations:** ^1^ School of Biological Sciences University of Nebraska‐Lincoln Lincoln Nebraska USA; ^2^ Department of Health and Environmental Sciences Xian Jiaotong‐Liverpool University Suzhou China

**Keywords:** annual variation, drought, functional trait, grassland, intraspecific trait variation, NutNet, nutrients

## Abstract

Effective application of functional trait approaches to ecological questions requires understanding the patterns of trait variation within species as well as between them. However, few studies address the potential for intraspecific variation to occur on a temporal basis and, thus, for trait‐based findings to be contingent upon sampling year. To quantify annual variation in the functional traits of grassland plant species, we measured specific leaf area, leaf dry matter content, plant height, and chlorophyll content in 12 shortgrass prairie plant species. We repeated these measurements across 4 years, both in long‐term nitrogen addition plots and in corresponding control plots. Three of the four traits showed significant year‐to‐year variation in a linear mixed model analysis, generally following a pattern of more acquisitive leaf economics spectrum traits in higher rainfall years. Furthermore, two of the measured traits responded interactively to nitrogen addition and sampling year, although only one, leaf dry matter content, showed the expected pattern of stronger nitrogen responses in high rainfall years. For leaf dry matter content and specific leaf area, trait responses to sampling year were larger than responses to the nitrogen addition treatment. These findings illustrate that species' functional traits can respond strongly to environmental changes across years, and thus that trait variation in a species or community is likely to extend beyond the values and patterns observed in any single year.

## INTRODUCTION

Functional trait measurements have emerged as a powerful tool in addressing a broad range of ecological questions. Considered at the species level, functional traits can predict the distributions of organisms and the interactions between them. Considered at the community level, these same traits can predict a wide range of ecosystem functions (Funk et al., 2017). As such patterns are not restricted to particular taxa, analyses based on functional traits may prove a more powerful avenue toward finding generalizable ecological rules than “nomenclatural”, or species‐based comparisons (McGill et al., 2006).

Functional traits' associations with ecological trade‐offs suggest that organisms with particular combinations of traits should thrive under corresponding sets of environmental conditions. The leaf economics spectrum in plants, described at a biogeographical level by Wright et al. (2004) and quickly extended to smaller‐scale comparisons (Garnier et al., 2007; Gross et al., 2007; McIntyre & Lavorel, 2007), is arguably the best studied example of such a pattern: under cool, dry and nutrient‐poor conditions, plants tend to produce slow growing, long‐lived leaves. This strategy and its associated trait values, including low nitrogen content, low specific leaf area (SLA), and high leaf dry matter content (LDMC) are described as conservative, while the opposing strategy and associated trait values, found under warmer, wetter, and more nutrient‐rich conditions, are described as acquisitive.

Applications of functional traits to ecological questions depend largely on trait comparisons at the species level. For these purposes, species average trait values may be either calculated locally or drawn from global databases such as TRY (Kattge et al., 2011). However, such comparisons are complicated by the large portion of functional trait variation that occurs within species. According to a meta‐analysis by Siefert et al. (2015), intraspecific trait variation makes up 25% of functional trait variation within plant communities and 32% of the functional trait variation between communities. Whether through plastic environmental response (Reich et al., 1996; Vitasse et al., 2010), selection on genetic variants, or a combination of both processes (Olsen et al., 2013), intraspecific variation can be expected to produce a pattern of individual‐level trait values better suited to local conditions than species‐level averages. For instance, nutrient addition experiments (La Pierre & Smith, 2015; Siefert & Ritchie, 2016; Tatarko & Knops, 2018) show that individuals of a given species exhibit more acquisitive leaf economics spectrum traits under nutrient enrichment treatments than conspecifics under ambient conditions. Similarly, intraspecific trait variation often corresponds to patterns of water availability or precipitation (Guo et al., 2017; Nunes et al., 2017; Olsen et al., 2013; Yue et al., 2019).

As environmental conditions vary across years, functional trait measurements may be expected to vary on an annual basis, even when sampling areas and methodology remain constant. A study by Garnier et al. (2001) focused on quantifying trait variation across temporal and spatial scales, and several subsequent studies have documented trait differences between pairs of study years (Chen et al., 2019; Luo et al., 2019; Tatarko & Knops, 2018). Although the causes of such changes can rarely be directly tested, climatic variation appears the most likely cause.

Annual variation in species' traits may play an important role in their interactions with one another and with the broader environment. If not properly accounted for, however, this variation could undermine the assumptions trait‐based analysis depends upon (Shipley et al., 2016), resulting in mistaken extrapolations and causing important community dynamics to be overlooked. Analyses extrapolating from a single set of species‐level trait measurements would, for instance, fail to detect the plastic effects that contribute to community level change (La Pierre & Smith, 2015) if that change occurred on a temporal basis. Even explicit measurements of trait variability could be substantially underestimated if derived based on sampling at a single point in time, and comparisons of trait values between sites could be contingent on sampling conditions. Temporal variation might also have interactive effects with other variables of interest, analogous to biomass effects in water/nutrient colimited systems (Wang et al., 2017).

More detailed knowledge of year‐to‐year trait variation will allow us to better understand how much variation is undetected in single‐year sampling and how likely such variation is to alter ecological processes and analyses. Currently, however, studies of year‐to‐year intraspecific variation are rare, with existing multi‐year studies considering only 2 years of trait data (Chen et al., 2019; Garnier et al., 2001; Tatarko & Knops, 2018) or focusing their data collection and analysis on community‐weighted, rather than intraspecific change (Chen et al., 2019; Luo et al., 2019; Tatarko & Knops, 2018). A larger temporal sample should allow for variability estimates less sensitive to the individual years chosen, as well as more detailed comparisons of trait differences to possible explanatory factors. We therefore chose to monitor intraspecific trait variation across four study years in a North American grassland site. Through this sampling, we sought to answer the following ecological questions:

1. How variable are plant species' functional traits across years? Based on existing two‐year studies, we predicted that trait variation between years could approach, or even exceed, the level of variation seen between fertilized and unfertilized study plots.
2. Does intraspecific trait variation correspond to environmental variation? We anticipated that trait values would respond to growing season precipitation, with plant species displaying more conservative trait values under low rainfall conditions.
3. Do trait responses to nutrient availability depend on sampling year? Based on productivity responses observed at our study site (Wang et al., 2017), we expected that nitrogen addition and study year would affect species' traits interactively, with stronger nitrogen effects apparent in high rainfall years.

## METHODS

To address these questions, we conducted repeated surveys of functional traits in study plots at Cedar Point Biological Station in western Nebraska, USA (41°12′ N, 101°38′ W). These study plots occur in shortgrass prairie on bluffs overlooking Lake Ogallala and were established in 2007 in association with the Nutrient Network global collaboration (Borer et al., 2014). The site shows a distinct pattern of resource limitation by water and nitrogen. In dry years, productivity shows no significant response to nutrient addition. In wet years, overall productivity increases, and nitrogen addition leads to a further productivity increase, driven primarily by responsive annual species. Thus, productivity appears to be limited by water in dry years but by nitrogen in wet years (Wang et al., 2017). This pattern creates ideal circumstances for testing effects of both nutrient and water availability, as well as any interactions between them. Over the course of our study, 2 years, 2017 and 2020 had below average growing season precipitation (179 and 154 mm respectively, between 1 April and 30 June, compared to an average of 206 mm, Appendix S1: Figure S1), while the other two had above average precipitation (322 mm in 2018 and 214 mm in 2019). Previous trait sampling at this site, focused on community‐level variation (Tatarko & Knops, 2018), shows functional trait responses to nitrogen, as well as temporal variation over two study years.

### Sampling methods

To assess year‐to‐year intraspecific trait variation, we surveyed plants within 12 5 × 5 m study plots, repeating our sampling in each year from 2017 to 2020. We surveyed only control and nitrogen addition plots, as past research has shown other nutrients do not alter functional traits (Tatarko & Knops, 2018) or productivity (Wang et al., 2017) at this site. Nitrogen was applied to the addition plots at a rate of 10 gN m^−2^ year^−1^ in the form of time‐release urea, and each addition plot was associated with a control plot in one of the site's six experimental blocks.

For our trait sampling, we selected 12 species common at our site and spanning a range of functional groups (Table 1). For each species‐plot combination, we measured SLA, LDMC, maximum height, and chlorophyll content. While some species were absent in specific plots or years, we were able to collect 383 sets of trait measurements across the four study years. To produce plot‐level height measurements, we measured three individuals per species‐plot combination and averaged their measured heights. For measurements of the remaining traits, we collected aggregated leaf samples, drawing from individuals distributed throughout the study plots. All leaves collected were fully expanded, non‐senesced, and showed minimal herbivore/pathogen activity. Where possible, we collected enough leaves to constitute 1 g of total fresh biomass. We measured chlorophyll content of three leaves per species‐plot sample using a CCM‐300 Chlorophyll Content Meter (Opti‐Science, Hudson, New Hampshire, USA), averaging three readings per leaf to improve measurement precision. As with height, we averaged these three measurements to generate a plot‐level measurement.

**TABLE 1:** Study species: 12 species were included in trait sampling, representing a range of functional groups.

Species	Functional group	Lifespan	Photosynthetic pathway	Provenance	Mean percent cover
*Artemisia filifolia*	Shrub	Perennial	C3	Native	3.6
*Artemisia frigida*	Forb	Perennial	C3	Native	5.4
*Bouteloua gracilis*	Graminoid	Perennial	C4	Native	7.9
*Bromus tectorum*	Graminoid	Annual	C3	Introduced	28
*Carex filifolia*	Graminoid	Perennial	C3	Native	14
*Dichanthelium oligosanthes*	Graminoid	Perennial	C3	Native	2.0
*Helianthus annuus*	Forb	Annual	C3	Native	1.8
*Hesperostipa comata*	Graminoid	Perennial	C3	Native	9.8
*Lithospermum incisum*	Forb	Perennial	C3	Native	0.27
*Pascopyrum smithii*	Graminoid	Perennial	C3	Native	2.2
*Psoralidium tenuiflorum*	Legume	Perennial	C3	Native	0.57
*Sphaeralcea coccinea*	Forb	Perennial	C3	Native	0.39

*Note*: Cover values represent mean abundance from 2017 to 2020 across all cedar point nutrient network plots.

To compute SLA and LDMC, we measured the fresh mass of each sample to the nearest 0.01 g and measured the total leaf area using an LI‐3000A leaf area meter (LI‐COR Lincoln, Nebraska, USA). For species with leaves too narrow to be accurately measured in this manner (*Artemisia filifolia*, *Artemisia frigida*, *Bromus tectorum*, and *Carex filifolia*), we instead imaged the leaves in a flat‐bed scanner with a resolution of 600 dpi and calculated total area using ImageJ software (NIH, Bethesda, Maryland, USA). We then dried the samples in a forced air oven at 60°C for a minimum of 48 h before making dry mass measurements. For each species‐plot combination, we calculated SLA as total leaf area divided by total dry mass and LDMC as total dry mass divided by total fresh mass.

### Data analysis

To test for year and nutrient effects on species' functional traits, we developed linear mixed effects models in R version 3.6.2 (R Core Team, 2019), treating sampling year and nutrient addition as fixed effects. We treated species as a random effect, as our interest was in overall patterns of intraspecific variation, rather than the responses of individual species. We likewise treated experimental block as a random effect, although we omitted this factor from the multivariate and chlorophyll models, where its effect was extremely small and prevented model convergence. Height and SLA were log transformed to better conform to the assumptions of linear modeling, while chlorophyll and LDMC were analyzed with their original units.

To prevent the per‐family Type I error rate from increasing due to multiple comparisons with the four response traits, we first conducted MANOVA testing of year and nutrient effects on the full array of functional traits and then applied an adjusted alpha of 0.05/(4–1) = 0.0167 for subsequent univariate tests (Frane, 2015). In order to incorporate species random effects in multivariate model fitting, we used the MCMCglmm function (Hadfield, 2010). We evaluated the significance of year, nutrient and interactive effects by comparing Deviance Information Criterion (DIC) values of models including and omitting each of these effects. Contingent on the significance of this multivariate analysis, we proceeded to fit models for each individual functional trait, using the lme4 package (Bates et al., 2015). For these models, we applied ANOVA tests to evaluate year, nutrient and interactive effects, followed by Tukey tests (using the multcomp R package) for post‐hoc comparisons.

## RESULTS

The multivariate analysis provided evidence of an interactive effect of sampling year and nitrogen treatment on the measured functional traits, with the interactive model outperforming an additive version (ΔDIC = 8.7). Both additive and interactive nutrient/year models outperformed models incorporating only a single fixed effect predictor variable (Table 2), providing strong support for further analysis of these variables' main and interactive effects on individual traits, given the use of an adjusted alpha value (Bird & Hadzi‐Pavlovic, 2014).

**TABLE 2:** Multivariate model comparison.

Model	Deviance	Effective parameters	DIC	ΔDIC
Year × nitrogen	3684.0	31.9	3747.8	0
Year + nitrogen	3695.6	30.5	3756.5	8.7
Year	3707.6	30.0	3767.6	19.7
Nitrogen	3801.3	29.0	3859.2	111.3

*Note*: Each linear mixed model incorporates year and/or nitrogen addition as fixed effects with species identity as a random effect and the four measured functional traits as response variables. For each model, deviance, effective parameters, and deviance information criterion are calculated using the MCMCglmm function (Hadfield, 2010), with ΔDIC values calculated by subtracting the Deviance Information Criterion (DIC) value of the most predictive model, which includes an interactive effect of year and nutrient treatment.

**FIGURE 1:**
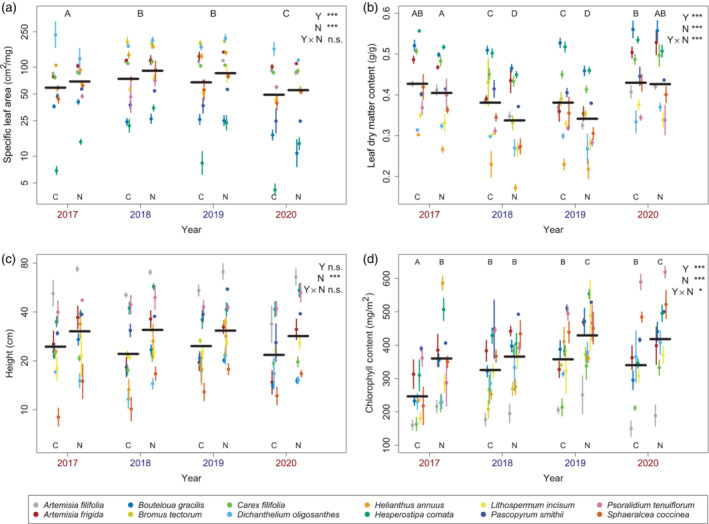
Trait responses to year and nutrient treatment. Colored points represent species means for each treatment‐year combination, with the associated vertical lines showing standard errors of those means. Black, horizontal lines represent the expected values for each treatment‐year combination, generated by linear mixed modeling (Appendix S1: Table S1). Standard errors for these overall means are not shown, as they largely reflect the variation accounted for by the models' species terms. Overall significance of year (Y), nutrient (N), and interactive (Y × N) effects is indicated at the top right of each panel, with * indicating *p* values below the adjusted alpha value of 0.0167 and *** indicating *p* values less than 0.001. For traits with significant year effects or treatment‐year interactions, significant differences between individual years or treatment‐year combinations (*p* < 0.05 in a Tukey post‐hoc comparison) are indicated by letters above the plotted points. Blue text indicates years with above average rainfall, with red text indicating below average rainfall. n.s., non significant.

In the analyses of individual traits, the largest SLA differences were due to species identity (with a variance of 0.539 log units), but highly significant responses could be attributed to both sampling year (χ^2^ = 82.8, df = 3, *p* < 0.001; Appendix S1: Table S1) and nutrient treatment (χ^2^ = 24.4, df = 1, *p* < 0.001). Leaves measured in 2018 and 2019 showed higher SLA values than those measured in 2017 and 2020, with a difference of 0.45 log units between the most distinct years (2018 and 2020), representing a difference between 49.0 and 74.1 cm^2^/g after reverse transformation (see Appendix S1: Table S2 for full post‐hoc comparisons). These patterns align with weather data, in that 2017 and 2020 had below average growing season precipitation while 2018 and 2019 had above average precipitation. Nitrogen addition led to an increase in species' SLA values by only 0.16 log units, representing an increase from 58.9 to 69.1 cm^2^/g. Contrary to expectations, there was no significant interaction between nutrient treatment and sampling year (χ^2^ = 1.42, df = 1, *p* = 0.700; Figure 1a).

LDMC measurements showed a similar response pattern to SLA, with more acquisitive values recorded in high rainfall years (2018 and 2019), and in nutrient addition plots. In contrast to SLA, nutrient effects on LDMC varied significantly from year to year (χ^2^ = 17.7, df = 3, *p* = 0.001; Figure 1b; Appendix S1: Table S1), with large nitrogen effects observed in wet years (4.4 and 3.9 percentage points in 2018 and 2019, respectively) and smaller, non‐significant differences observed in dry years. Among control plots, the largest year to year difference, 4.9 percentage points, occurred between 2019 and 2020, while for nitrogen plots, the largest year to year difference was 8.9 percentage points, between 2018 and 2020 (see Appendix S1: Table S3 for full post hoc comparisons).

Height measurements differed from SLA and LDMC in that trait differences between years were not statistically significant (χ^2^ = 7.55, df = 3, *p* = 0.056; Appendix S1: Table S1). Plant heights did increase significantly with nutrient addition (χ^2^ = 85.7, df = 1, *p* < 0.001), from an average of 24.3 to 30.4 cm after reverse transformation. There was no significant nutrient‐year interaction (χ^2^ = 3.19, df = 3, *p* = 0.363) (Figure 1c).

Chlorophyll, like LDMC, responded interactively to nutrient addition and year (χ^2^ = 10.3, df = 3, *p* = 0.0164; Figure 1d; Appendix S1: Table S1). In this case, however, the largest nitrogen effect occurred in 2017, increasing chlorophyll content by 113.2 mg/m^2^, while the smallest nitrogen effect, a non‐significant difference of 40.2 mg/m^2^, occurred in 2018. Leaves sampled in 2017 showed the lowest chlorophyll levels overall, while those sampled in 2019 showed the highest levels, with an average difference of 110.9 mg/m^2^ for control plots and 69.2 mg/m^2^ for nitrogen addition plots sampled in these years (see Appendix S1: Table S4 for full post‐hoc comparisons).

**FIGURE 2:**
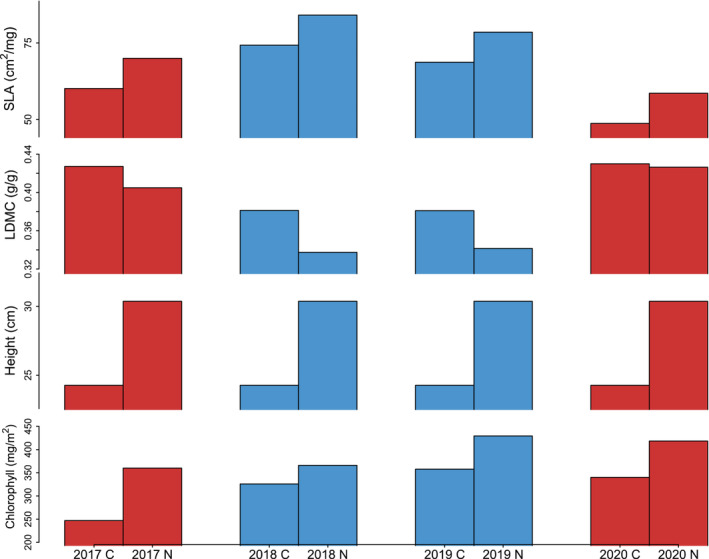
Modeled trait responses to sampling year and nitrogen addition. Bars represent expected mean trait values across all species, based on the linear mixed model for each trait, with non‐significant effects omitted from calculations. Red bars indicate low rainfall years, with blue bars indicating high rainfall years.

**FIGURE 3:**
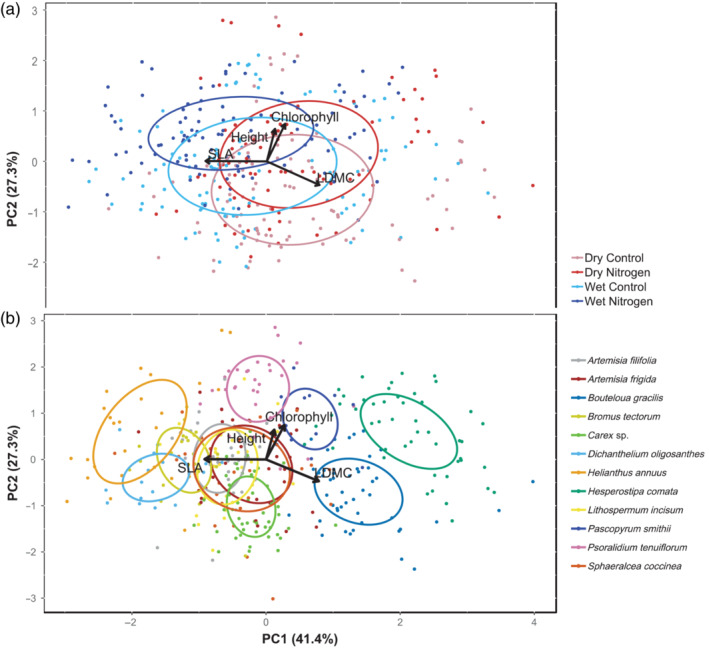
Principal component analysis of trait variation. In panel (a), data points are grouped by nutrient treatment and sampling year, with years combined for clarity into wet years (2018 and 2019) and dry years (2017 and 2020). In panel (b), data points are grouped by species. Each point represents a plot‐level trait measurement, while ellipses represent standard errors around each group's midpoint.

Overall, three of four traits shifted toward more acquisitive values in high rainfall years and, with the exception of chlorophyll in 2020, showed more conservative values in dry years (Figure 2). While functional groups differed in the strength of some effects, these differences were not consistent from trait to trait, and accounting for them did not alter the overall significance of year and nitrogen effects (Appendix S1: Figure S3, Table S5). Across traits, responses to sampling year were most consistent for SLA and LDMC, the study traits most closely associated with the leaf economics spectrum. Height, and to a lesser extent chlorophyll content responded more clearly to nutrient addition than to sampling year, a pattern illustrated by principal components based visualization of the data (Figure 3).

## DISCUSSION

These results illustrate that variation across years is an important component of intraspecific variation in plant functional traits. Three of our four studied traits showed significant year‐to‐year variation, and for SLA and LDMC, annual variation exceeded variation in response to nitrogen addition, as well as annual change observed in previous, shorter‐term studies (Garnier et al., 2001; Tatarko & Knops, 2018). We also observed that annual variation in these traits was consistent with expected water availability responses, with more acquisitive trait values occurring in high rainfall years. Finally, while interactions between year and nitrogen effects were less consistent in strength and direction than overall annual changes, trait responses to nitrogen did vary across years for both LDMC and chlorophyll content.

Comparisons of year and nutrient effect sizes are particularly striking in that this study's year‐to‐year comparisons reflect only ambient levels of variation, while its nitrogen effects reflect a decade long experimental manipulation. Although effect sizes are presumably site dependent, these results do suggest that annual intraspecific variation may often exceed variation driven by natural, or ambient anthropogenic, levels of nutrient enrichment. Nitrogen deposition rates in western North America are expected to remain below 2 g m^−2^ year^−1^ through 2050 (Galloway et al., 2004), while our addition treatment added nitrogen at five times this rate. Effects of annual variation could be further enhanced in settings with greater annual variation in conditions. Such sites, and those with weaker nutrient limitation, could see annual effects exceed nutrient effects by substantial margins. This would result in a significant underestimates of intraspecific trait variation when such estimates are based on sampling conducted in a single year.

Although we were not able to directly test mechanisms underlying the observed annual trait variation, the observed patterns match our expectations for a scenario where trait responses are driven by rainfall. The low SLA, high LDMC, and low chlorophyll content (indicative of low leaf N) observed in the drier conditions of 2017 and 2020 are all associated with the conservative leaf economics spectrum strategies we would expect to observe in resource‐poor conditions (Garnier et al., 2007; Gross et al., 2007; McIntyre & Lavorel, 2007; Wright et al., 2004), a pattern also consistent with the results observed in shorter‐term studies (Chen et al., 2019; Garnier et al., 2001; Tatarko & Knops, 2018). More direct tests of this causal mechanism would, however, be valuable, as annual effects on ecological variables may stem from other factors even in settings where precipitation plays an important role (Groves & Brudvig, 2019). Future analyses incorporating experimental rainfall manipulation will aid in uncovering what portion of annual trait variation can be attributed to precipitation. Longer‐term observational studies will also be valuable, generating data sets with sufficient sample size and precipitation variation for regression‐based analyses.

To the extent that annual trait variability is a response to precipitation, these measurements will be valuable in projecting changes to trait values under future climatic changes. In western North American grasslands, global climate change is expected to result in more frequent and intense drought (Cook et al., 2015; Dai, 2013). While the most extreme future conditions may be beyond the range of current measurements, current studies can nonetheless provide valuable insight into species' capacities for intraspecific change. Similar approaches could be applied in settings where temperature or other environmental variables drive trait responses.

A surprising aspect of our results is that, while we expected to find stronger nitrogen effects in high rainfall years, corresponding to the productivity effects observed by Wang et al. (2017), our data did not consistently show such a pattern. The effects of nitrogen addition and sampling year on height and SLA instead followed a purely additive pattern. Chlorophyll content did show an interactive response to these variables, but did not follow the expected pattern, instead showing a stronger nitrogen response in one of the two low rainfall years, a weaker nitrogen response in one of the two high rainfall years, and intermediate nitrogen responses in the two remaining years. Only LDMC showed the expected pattern of larger nitrogen effects corresponding to high rainfall conditions. The divergence of these traits' patterns of variation from one another and from the site's pattern of productivity response (Wang et al., 2017) emphasizes that trait values and productivity can both respond to complex arrays of variables and thus may not always be correlated as expected.

In those cases where traits do respond additively to nitrogen and sampling year, the lack of interaction, although unexpected, is potentially a valuable finding. In such cases, 1 year of trait sampling should be sufficient for the purpose of evaluating nutrient effects on functional traits. While extrapolation of such response patterns between sites will not always be reliable, and while multi‐year data will remain valuable in evaluating other study questions, a single sampling year approach holds obvious advantages in speed and in required labor. Confirming the circumstances under which such an approach is likely to yield representative findings would therefore be of great benefit in planning future research.

Analysis of annual variation may substantially improve ecological predictions in many contexts, including species and community responses to climate change. A large scale analysis of tundra sites (Bjorkman et al., 2018) illustrates the potential for intraspecific variation to shift functional trait averages to a greater degree than species turnover alone would predict. Such shifts may substantially alter ecosystem function parameters, and annual variation will influence estimates of intraspecific variability in evaluating such scenarios. Intraspecifically driven changes to community traits may also have consequences beyond the communities in question. For instance, accounting for temperature‐induced changes to boreal forests' needle lifespan can improve productivity and carbon cycling estimates in climate models (Reich et al., 2014). Changes to productivity or decomposition rates, mediated by leaf economics spectrum traits, could have similar effects, and incorporating year‐to‐year variability into estimates of potential intraspecific variation could strongly influence estimates of these effects' magnitude.

Intraspecific trait variation also holds substantial implications for species coexistence. Most trait‐based analyses of coexistence, such as those proposed by Adler et al. (2013), rely on species‐level trait values, which may uncover informative patterns (Catford et al., 2019; Kraft et al., 2008; Stubbs & Wilson, 2004) but does risk misrepresenting trait differentiation by failing to account for overlap between species' trait ranges (Violle et al., 2012). Interactions between individuals will ultimately be determined by the traits of those individuals rather than by species averages, and accounting for intraspecific trait variation allows more precise detection of niche differentiation in trait‐based studies (Lasky et al., 2014; Paine et al., 2011; Siefert, 2012). Incorporating annual variation could provide further precision.

Intraspecific variation can also act itself as a mechanism promoting or inhibiting coexistence. Mathematical modeling suggests intraspecific variation may aid coexistence in specific scenarios (Crawford et al., 2019; Uriarte & Menge, 2018), but in general, intraspecific variation can be expected to interfere with coexistence by increasing interspecific competition and decreasing intraspecific competition (Barabás & D'Andrea, 2016; Hart et al., 2016; Turcotte & Levine, 2016), thereby inhibiting stabilizing mechanisms of coexistence, as defined by Chesson (2000). Whether responses to annual variation enhance such barriers to coexistence will depend on the precise nature of species' responses. If all species respond in the same manner, trait differences between species will be maintained, and changes to coexistence dynamics should be minimal. Such responses are often discussed in terms of consistency of rank order, and several studies have found these orders to be relatively consistent, even as environmental change shifts species' traits (Fajardo & Siefert, 2016; Garnier et al., 2001; Kazakou et al., 2014). However, even shifts that retain rank order could substantially impact species' niche overlap with one another and thus their coexistence dynamics. Greater species‐level analysis of annual trait effects could help predict the frequency of such changes.

In summary, we find that annual variability is a major component of intraspecific trait variation and is therefore likely to have a substantial impact on trait‐based ecological interactions, as well as on the results of trait‐based research. The latter effect may be particularly pertinent to studies aimed at quantifying populations' trait variation or attempting to extrapolate trait values across time or space. While annual trait variation appears correlated with water availability at our study site, further experimental manipulation will be needed to clearly establish the relevant causal mechanisms. It is also likely that annual intraspecific trait responses will, to some degree, vary with the species, trait, and geographic location considered. Studies evaluating trait variation in other settings would be of great value in determining which patterns of year‐to‐year trait variation can be extrapolated across sites and which patterns are more contingent upon experimental context. Such research could be particularly valuable at long‐term research sites where many future trait‐based studies could be informed by locally focused analyses. While it is neither feasible nor necessary that all trait‐based studies measure annual variability, it is critical that the potential effects of such variation be considered during the design and interpretation of trait‐based experiments.

## CONFLICT OF INTEREST

The authors declare no conflict of interest.

## Supporting information


Appendix S1:

## Data Availability

Data and R code (Wheeler, 2022) are available in Figshare at https://doi.org/10.6084/m9.figshare.16958818.
