# Supplementary material for: Functional traits' annual variation exceeds nitrogen‐driven variation in grassland plant species
Source: Ecology. 2022 Dec 7;104(2):e3886. doi: 10.1002/ecy.3886 (PMC10078297; doi:10.1002/ecy.3886)
Supplement: Supplementary file 1 — Appendix S1: [file ECY-104-0-s001.pdf]

**Ecology**

# Functional traits' annual variation exceeds nitrogen-driven variation in grassland plant species

**George R. Wheeler, Chad E. Brassil and Johannes M.H. Knops**

**Supplemental Materials: Appendix S1**

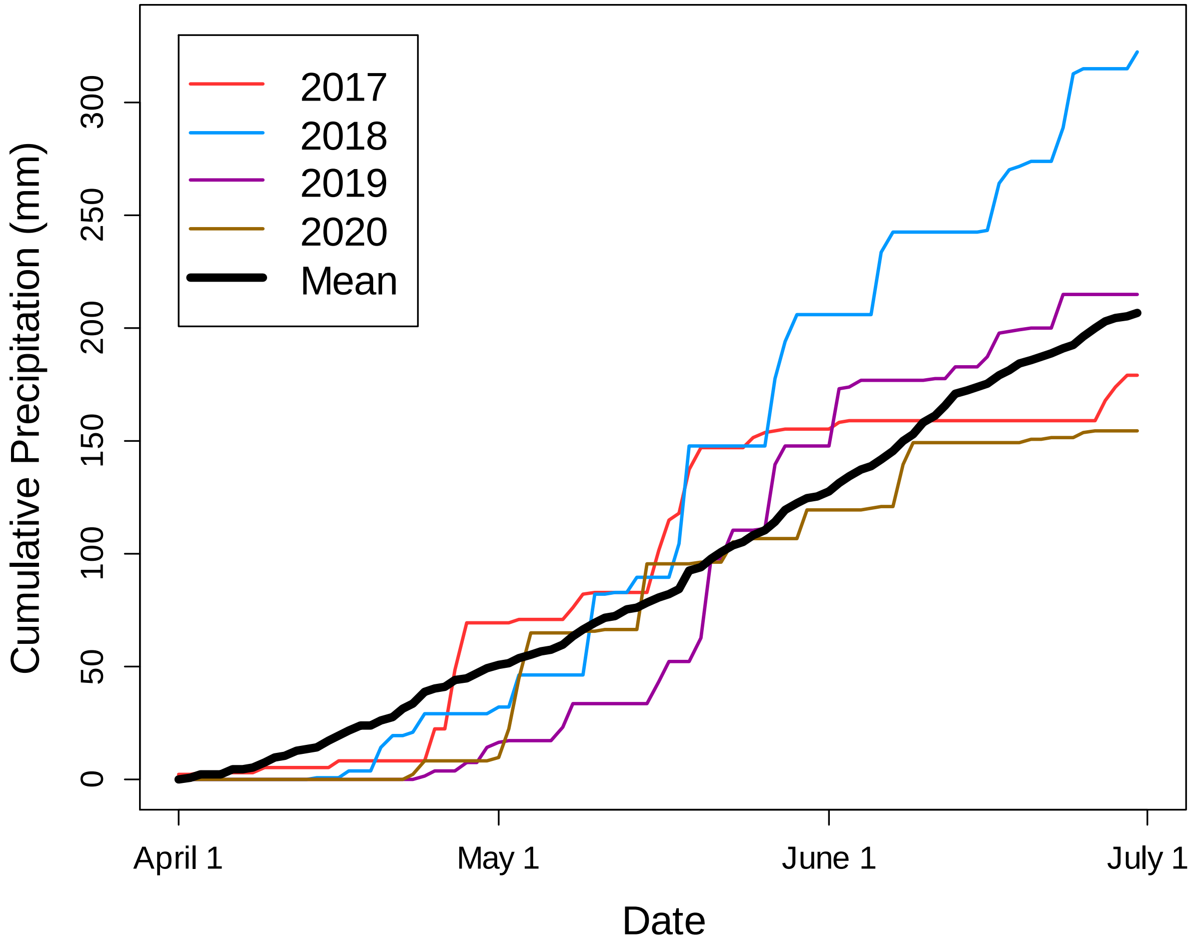


**Figure S1.** Cumulative precipitation from April 1, shown in each of the four study years, along with mean accumulation over all years since weather station establishment in 1997. Note below average precipitation by season end in 2017 and 2020 and above average precipitation in 2018 and 2019.

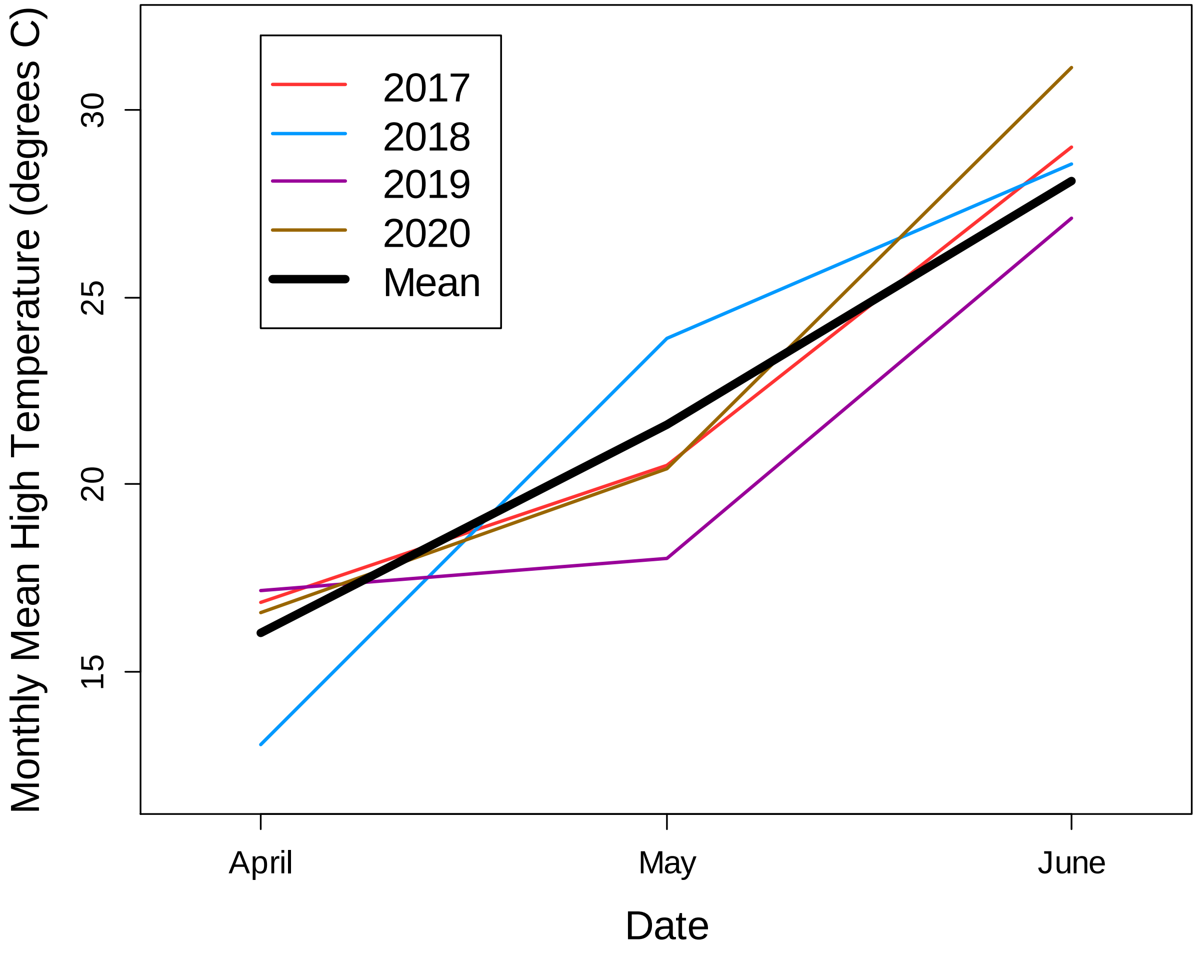


**Figure S2.** Monthly mean of daily high temperatures in the growing season of each study year, shown with mean conditions since weather station establishment in 1997.

**Table S1:** Linear mixed model output for single trait analyses. See methods for model descriptions.

| | | | **Fixed Effects** | | | | **Random Effects** | |
|---|---|---|---|---|---|---|---|---|
| **Specific Leaf Area** | | ***Nutrient*** | ***Year*** | *Nutrient*Year* | | **Species** | **Block** | **Residual** |
| | $\chi2$ | **24.3745** | **82.8156** | 1.4226 | Standard Deviation | 0.73 (n=12) | 0.07 (n=6) | 0.36 |
| | df | **1** | **3** | 3 | | | | |
| | p | **7.931e-07** | **< 2.2e-16** | 0.7002 | | | | |
| **Leaf Dry Matter Content** | | ***Nutrient*** | ***Year*** | ***Nutrient*Year*** | | **Species** | **Block** | **Residual** |
| | $\chi2$ | **56.647** | **247.895** | **17.670** | Standard Deviation | 0.078 (n=12) | 0.007 (n=6) | 0.036 |
| | df | **1** | **3** | **3** | | | | |
| | p | **5.215e-14** | **< 2.2e-16** | **0.0005144** | | | | |
| **Height** | | ***Nutrient*** | ***Year*** | *Nutrient*Year* | | **Species** | **Block** | **Residual** |
| | $\chi2$ | **85.7103** | 7.5490 | 3.1891 | Standard Deviation | 0.42 (n=12) | 0.08 (n=6) | 0.28 |
| | df | **1** | 3 | 3 | | | | |
| | p | **< 2e-16** | 0.05631 | 0.36338 | | | | |
| **Chlorophyll** | | ***Nutrient*** | ***Year*** | ***Nutrient*Year*** | | **Species** | **Block** | **Residual** |
| | $\chi2$ | **81.244** | **73.577** | **10.270** | Standard Deviation | 80.0 (n=12) | **-** | 78.9 |
| | df | **1** | **3** | **3** | | | | |
| | p | **< 2.2e-16** | **7.314e-16** | **0.0164** | | | | |

**Table S2** Tukey post-hoc comparisons of SLA across sampling years. See Table S1 for primary analysis.

| Years | Estimated Difference | Standard Error | z | p |
|---|---|---|---|---|
| **2017:2018** | **0.25256** | **0.05243** | **4.817** | **< 0.001** |
| **2017:2019** | **0.17334** | **0.0525** | **3.302** | **0.00513** |
| **2017:2020** | **-0.20124** | **0.05393** | **-3.732** | **< 0.001** |
| 2018:2019 | -0.07923 | 0.04984 | -1.59 | 0.3843 |
| **2018:2020** | **-0.4538** | **0.05344** | **-8.491** | **< 0.001** |
| **2019:2020** | **-0.37458** | **0.05343** | **-7.011** | **< 0.001** |

**Table S3** Tukey post hoc comparisons of LDMC across nitrogen treatments and sampling years. See Table S1 for primary analysis.

| Years/ Treatments | Estimated Difference | Standard Error | z | p |
|---|---|---|---|---|
| 2017C:2017N | -0.02231 | 0.007706 | -2.894 | 0.073 |
| **2017C:2018C** | **-0.04599** | **0.00727** | **-6.326** | **<0.001** |
| **2017C:2018N** | **-0.08974** | **0.007464** | **-12.024** | **<0.001** |
| **2017C:2019C** | **-0.04622** | **0.007203** | **-6.418** | **<0.001** |
| **2017C:2019N** | **-0.08562** | **0.007548** | **-11.342** | **<0.001** |
| 2017C:2020C | 0.00276 | 0.007347 | 0.376 | 1 |
| 2017C:2020N | -0.00081 | 0.007895 | -0.103 | 1 |
| **2017N:2018C** | **-0.02368** | **0.007527** | **-3.146** | **0.0351** |
| **2017N:2018N** | **-0.06744** | **0.007638** | **-8.829** | **<0.001** |
| **2017N:2019C** | **-0.02392** | **0.00748** | **-3.197** | **0.0301** |
| **2017N:2019N** | **-0.06331** | **0.007719** | **-8.202** | **<0.001** |
| **2017N:2020C** | **0.025066** | **0.007674** | **3.266** | **0.0238** |
| 2017N:2020N | 0.021494 | 0.008129 | 2.644 | 0.1395 |
| **2018C:2018N** | **-0.04375** | **0.007168** | **-6.104** | **<0.001** |
| 2018C:2019C | -0.00024 | 0.006967 | -0.034 | 1 |
| **2018C:2019N** | **-0.03963** | **0.007245** | **-5.47** | **<0.001** |
| **2018C:2020C** | **0.048748** | **0.007248** | **6.726** | **<0.001** |
| **2018C:2020N** | **0.045177** | **0.00778** | **5.807** | **<0.001** |

Table S3 continued

| Years/ Treatments | Estimated Difference | Standard Error | z | p |
|---|---|---|---|---|
| **2018N:2019C** | **0.043519** | **0.007108** | **6.122** | **<0.001** |
| 2018N:2019N | 0.004126 | 0.007307 | 0.565 | 0.9992 |
| **2018N:2020C** | **0.092502** | **0.007438** | **12.436** | **<0.001** |
| **2018N:2020N** | **0.088931** | **0.007912** | **11.24** | **<0.001** |
| **2019C:2019N** | **-0.03939** | **0.007189** | **-5.48** | **<0.001** |
| **2019C:2020C** | **0.048984** | **0.007175** | **6.827** | **<0.001** |
| **2019C:2020N** | **0.045413** | **0.00772** | **5.883** | **<0.001** |
| **2019N:2020C** | **0.088377** | **0.007525** | **11.745** | **<0.001** |
| **2019N:2020N** | **0.084805** | **0.007991** | **10.613** | **<0.001** |
| 2020C:2020N | -0.00357 | 0.00785 | -0.455 | 0.9998 |

**Table S4** Tukey post-hoc comparisons of chlorophyll content across nitrogen treatments and sampling years. See Table S1 for primary analysis.

| Years/ Treatments | Estimated Difference | Standard Error | z | p |
|---|---|---|---|---|
| **2017C:2017N** | **113.179** | **16.806** | **6.735** | **<0.01** |
| **2017C:2018C** | **78.735** | **15.869** | **4.962** | **<0.01** |
| **2017C:2018N** | **118.962** | **16.274** | **7.31** | **<0.01** |
| **2017C:2019C** | **110.927** | **15.717** | **7.058** | **<0.01** |
| **2017C:2019N** | **182.406** | **16.459** | **11.082** | **<0.01** |
| **2017C:2020C** | **93.279** | **16.038** | **5.816** | **<0.01** |
| **2017C:2020N** | **171.405** | **17.212** | **9.958** | **<0.01** |
| 2017N:2018C | -34.445 | 16.416 | -2.098 | 0.4143 |
| 2017N:2018N | 5.782 | 16.672 | 0.347 | 1 |
| 2017N:2019C | -2.252 | 16.314 | -0.138 | 1 |
| **2017N:2019N** | **69.226** | **16.846** | **4.109** | **<0.01** |
| 2017N:2020C | -19.9 | 16.742 | -1.189 | 0.9352 |
| **2017N:2020N** | **58.226** | **17.746** | **3.281** | **0.0227** |
| 2018C:2018N | 40.227 | 15.638 | 2.572 | 0.1657 |
| 2018C:2019C | 32.193 | 15.208 | 2.117 | 0.4028 |
| **2018C:2019N** | **103.671** | **15.807** | **6.558** | **<0.01** |
| 2018C:2020C | 14.544 | 15.816 | 0.92 | 0.9842 |
| **2018C:2020N** | **92.67** | **16.959** | **5.464** | **<0.01** |

Table S4 continued

| Years/ Treatments | Estimated Difference | Standard Error | z | p |
|---|---|---|---|---|
| 2018N:2019C | -8.034 | 15.505 | -0.518 | 0.9996 |
| **2018N:2019N** | **63.444** | **15.956** | **3.976** | **<0.01** |
| 2018N:2020C | -25.683 | 16.222 | -1.583 | 0.7601 |
| **2018N:2020N** | **52.443** | **17.263** | **3.038** | **0.0485** |
| **2019C:2019N** | **71.478** | **15.685** | **4.557** | **<0.01** |
| 2019C:2020C | -17.648 | 15.659 | -1.127 | 0.9509 |
| **2019C:2020N** | **60.478** | **16.831** | **3.593** | **<0.01** |
| **2019N:2020C** | **-89.127** | **16.413** | **-5.43** | **<0.01** |
| 2019N:2020N | -11.001 | 17.435 | -0.631 | 0.9985 |
| **2020C:2020N** | **78.126** | **17.125** | **4.562** | **<0.01** |

**Figure S3:** Trait responses to year and nutrient treatment by functional group. Colored points represent functional group means for each treatment-year combination with the associated vertical lines showing standard errors of those means. Graminoids show greater annual variability in SLA than non graminoids, but lower variability in LDMC. Nitrogen increases the height of non graminoids more strongly than graminoids, while chlorophyll content responds more strongly to nitrogen in graminoids than in non graminoids.

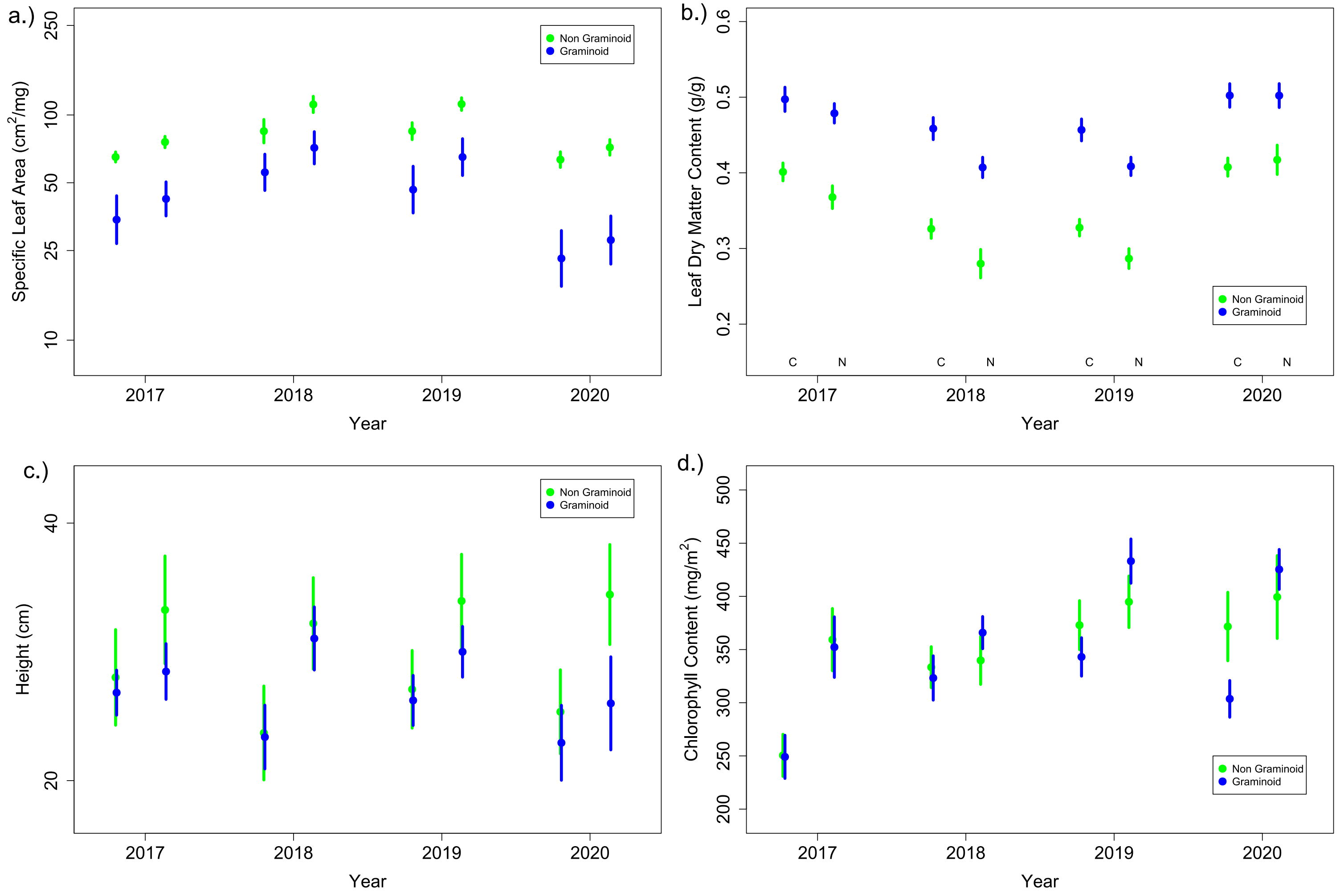

**Table S5:** Linear mixed model results with functional group effect.

| | Year | Nutrient | Functional Group | Year * Nutrient | Year * Functional Group | Nutrient * Functional Group | Year * Nutrient * Functional Group |
|---|---|---|---|---|---|---|---|
| **Specific Leaf Area** | | | | | | | |
| χ2 | **84.2771** | **25.4300** | 0.7802 | 1.0087 | **18.4061** | 0.4741 | 1.4748 |
| df | **3** | **1** | 1 | 3 | **3** | 1 | 3 |
| p | **< 2.2e-16** | **4.587e-07** | 0.3770897 | 0.7991563 | **0.0003627** | 0.4911296 | 0.6880893 |
| **Leaf Dry Matter Content** | | | | | | | |
| χ2 | **248.3399** | **57.0421** | **4.9976** | **19.1562** | **12.3881** | 0.8096 | 0.8907 |
| df | **3** | **1** | **1** | **3** | **3** | 1 | 3 |
| p | **< 2.2e-16** | **4.266e-14** | **0.0253830** | **0.0002538** | **0.0061654** | 0.3682372 | 0.8276781 |
| **Height** | | | | | | | |
| χ2 | 7.1830 | **77.7710** | 0.0929 | 2.9894 | 0.5648 | **13.9342** | 1.3081 |
| df | 3 | **1** | 1 | 3 | 3 | **1** | 3 |
| p | 0.0662894 | **< 2.2e-16** | 0.7605830 | 0.3932577 | 0.9044468 | **0.0001893** | 0.7272067 |
| **Chlorophyll Content** | | | | | | | |
| χ2 | **73.9876** | **82.6778** | 0.0019 | **11.0920** | 7.5426 | **6.5063** | 4.8960 |
| df | **3** | **1** | 1 | **3** | 3 | **1** | 3 |
| p | **5.971e-16** | **< 2.2e-16** | 0.96499 | **0.01124** | 0.05647 | **0.01075** | 0.17958 |
